# Modulation of Hypoxia-Induced Pulmonary Vascular Leakage in Rats by Seabuckthorn (*Hippophae rhamnoides* L.)

**DOI:** 10.1093/ecam/nep199

**Published:** 2010-09-15

**Authors:** Jayamurthy Purushothaman, Geetha Suryakumar, Dhananjay Shukla, Himani Jayamurthy, Harinath Kasiganesan, Rajesh Kumar, Ramesh Chand Sawhney

**Affiliations:** ^1^Defence Institute of Physiology and Allied Sciences, DRDO, Ministry of Defence, Timarpur, Delhi 110054, India; ^2^National Institute for Interdisciplinary Science and Technology, CSIR, Industrial Estate, Pappanamcode, Trivandrum, Kerala, India; ^3^Gazes Cardiac Research Institute, Medical University of South Carolina, Charleston, SC, USA

## Abstract

Cerebral and pulmonary syndromes may develop in unacclimatized individuals shortly after ascent to high altitude resulting in high altitude illness, which may occur due to extravasation of fluid from intra to extravascular space in the brain, lungs and peripheral tissues. The objective of the present study was to evaluate the potential of seabuckthorn (SBT) (*Hippophae rhamnoides* L.) leaf extract (LE) in curtailing hypoxia-induced transvascular permeability in the lungs by measuring lung water content, leakage of fluorescein dye into the lungs and further confirmation by quantitation of albumin and protein in the bronchoalveolar lavage fluid (BALF). Exposure of rats to hypoxia caused a significant increase in the transvascular leakage in the lungs. The SBT LE treated animals showed a significant decrease in hypoxia-induced vascular permeability evidenced by decreased water content and fluorescein leakage in the lungs and decreased albumin and protein content in the BALF. The SBT extract was also able to significantly attenuate hypoxia-induced increase in the levels of proinflammatory cytokines and decrease hypoxia-induced oxidative stress by stabilizing the levels of reduced glutathione and antioxidant enzymes. Pretreatment of the extract also resulted in a significant decrease in the circulatory catecholamines and significant increase in the vasorelaxation of the pulmonary arterial rings as compared with the controls. Further, the extract significantly attenuated hypoxia-induced increase in the VEGF levels in the plasma, BALF (ELISA) and lungs (immunohistochemistry). These observations suggest that SBT LE is able to provide significant protection against hypoxia-induced pulmonary vascular leakage.

## 1. Introduction

Acute exposure to hypoxia or high altitude induces a variety of illnesses like acute mountain sickness (AMS), high altitude pulmonary edema (HAPE) and high altitude cerebral edema (HACE). Among the high altitude illnesses, HAPE is the most common fatal manifestation accounting to most of the deaths [1]. Despite wide recognition of the clinical features and natural history of high-altitude-induced disorders, the exact pathogenesis involved remains speculative. Pulmonary hypertension, increased pulmonary capillary pressure and enhanced vascular permeability are the major factors for the development of HAPE [2, 3]. Hypoxia-induced oxidative stress plays a key role for the development of pulmonary hypertension and vascular leakage [4]. Earlier study by Bakonyi and Radak (2004) indicates that increased generation of reactive oxygen and nitrogen species may be responsible for hypoxia-induced increase in vascular permeability, which in turn may increase oxidative damage of lipids, proteins and DNA, besides decreasing the activity and effectiveness of the antioxidant enzyme system [5]. Therefore, supplementation with antioxidants may provide protection against hypoxia-induced oxidative stress and thus curtail high-altitude-induced maladies.

The hypoxic stress has also been suggested to elicit an inflammatory response, which chemotactically attracts alveolar macrophages and neutrophils resulting in release of inflammatory mediators that augment the fluid and albumin leakage [6].

Vascular permeability factor, also known as VEGF (vascular endothelial growth factor) has been demonstrated to be involved in the vascular basement-membrane destruction and angiogenesis [7–9]. VEGF has been shown to be up-regulated by hypoxic stress [10, 11] and its overexpression in the lung induces pulmonary vascular permeability, resulting in pulmonary edema [12]. In addition, the reactive oxygen intermediates have also been implicated to increase the VEGF levels [13, 14].

Seabuckthorn (SBT; *Hippophae rhamnoides* L., Elaegnaceae) is a unique and valuable plant currently being investigated for its medicinal values all over the world [15–17]. Studies from our laboratory using immune cells like rat spleenocytes, murine macrophages (J-774), human lymphocytes and C6 glioma cells have demonstrated that SBT leaves have a significant cytoprotective, immunomodulatory and antioxidant activity [18, 19]. Leaf drugs, which contain flavanoids, are implicated to increase the wound-healing effect after chemical burns and plain wounds [20]. The leaf extract (LE) was also found to provide protection against chromium-induced oxidative stress [21], besides having anti-inflammatory activity in adjuvant-induced arthritic animals [22]. The extract was also found to have significant antistress and adaptogenic activity when evaluated in cold-hypoxia-restrain animal model [23].

It is possible that administration of SBT LE may curtail hypoxia-induced transvascular permeability and thus may provide protection against high-altitude-induced maladies. Therefore, the present study was undertaken to evaluate efficacy of SBT LE in providing protection against hypoxic stress in animals exposed to acute hypobaric hypoxia in a decompression chamber.

## 2. Methods

### 2.1. Plant Material

SBT leaves were collected from the hilly region of Western Himalayas, India, in the month of August and dried under shade. The ethanobotanical identification of the plant was carried out by the Field Research Laboratory (Leh, India), where the voucher specimen of the plant material is preserved in the herbarium.

### 2.2. Plant Extract Preparation

The alcoholic LE was prepared using 70% ethanol as reported by Saggu et al. [23]. The powdered dry leaves were soaked in 70% ethanol (1 : 5 w/v) at room temperature (25 ± 1°C). After 24 h, the supernatant was decanted and the residue re-soaked in fresh 70% ethanol. The process was repeated four times for complete extraction. To avoid contamination, clean and sterile conditions were maintained during the extraction process. The supernatants were pooled, filtered through muslin cloth and stored in amber-colored bottles and centrifuged for 15 min at 8000 g, 4°C. The supernatant obtained after 15 min of centrifugation was frozen at −20°C and then lyophilized in a Heto Lyophilizer (HITOSICC, Heto-Holten A/S, Denmark). Lyophilized powder of the SBT leaf alcoholic extract was stored at −20°C in an airtight glass tube until further use. The crude yield of the lyophilized extract was determined gravimetrically by weighing the dried residues obtained out of known volumes of extract, extracted from known weight of dry leaves powder. One gram of dried SBT leaves produced 0.17 g of lyophilized SBT ethanolic extract powder.

### 2.3. Animals

**Figure 1:**
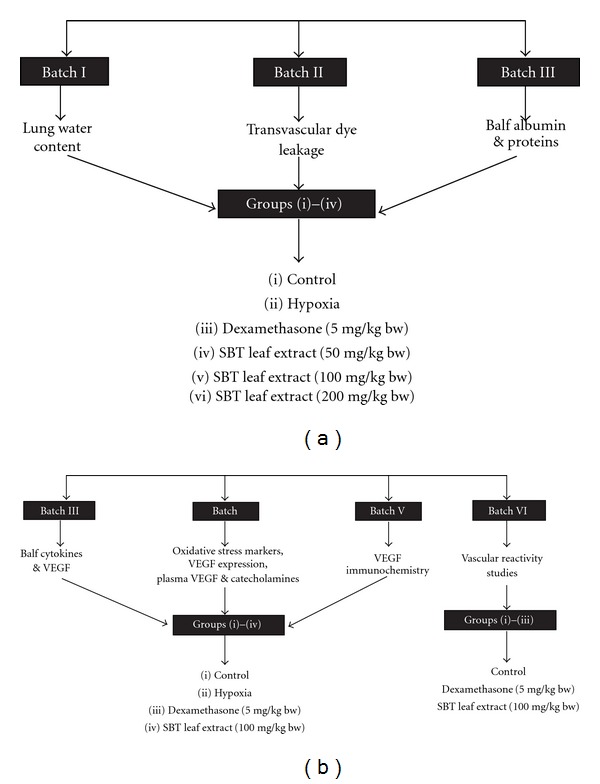
(a) Experimental design for the dose-response study to evaluate the efficacy of the extract in protection against hypobaric hypoxia-induced transvascular leakage in rats. The animals from group (ii) to (vi) were exposed to hypoxia of 9144 m for 5 h in a decompression chamber. Eight animals were used in each group. (b) Experimental design for effective dose studies. The animals from group (ii) to (vi) were exposed to hypoxia of 9144 m for 5 h in a decompression chamber except VIth batch. Eight animals were used in each group.

The study was performed in five batches of male Sprague-Dawley albino rats each weighing 170–200 g maintained at 24 ± 0.5°C with food and water *ad libitum*. The experimental protocol is shown in Figures 1(a) and 1(b). The study was approved by the Institute's Animal Ethical Committee and confirms to National guidelines on the care and use of laboratory animals.

#### 2.3.1. Dose-Response Study

The SBT extracts were diluted in sterile saline solution and different doses (50, 100 and 200 mg/kg b.w.) were administered orally using a gastric canula to determine their efficacy against hypoxia-induced increase transvascular permeability (Figure 1(a)). Initially, a dose-response study was carried out in three batches of animals comprising of six groups of eight animals each to investigate efficacy of SBT LE in modulating hypoxia-induced changes in water content and transvascular leakage in the lungs and albumin and protein levels in the bronchoalveolar lavage fluid (BALF).

#### 2.3.2. Efficacy of the Effective Dose of Leaf Extract

On the basis of these studies, an effective dose of SBT LE, which gave optimal protection against hypoxia-induced transvascular leakage, was used for further studies (Figure 1(b)).

### 2.4. Hypoxic Exposure

The different groups of animals received respective preparations as mentioned in Figure 1(a), orally, 12 h prior to exposure to hypoxia, with the help of a gastric canula, and the normoxic control group was maintained on saline. Similar method was followed for administration of dexamethasone (DEXA; 5 mg/kg body weight, bw) after dissolving in 1 mL normal saline. DEXA pretreated animals were taken as positive controls. The animals were exposed to 9144 m in a decompression chamber maintained at 24 ± 0.5°C (similar to the temperature at which the animals were normally housed) for 5 hours for studying the independent effect of hypoxic stress on transvascular leakage and its modulation by SBT. Airflow into the chamber was maintained at 2 L/min.

### 2.5. Lung Water Content

Wet-to-dry weight ratio was used as an index of tissue water content. After 5 h of hypoxic exposure the animals were anesthetized using ketamine (80 mg/Kg ip) and xylazine (20 mg/Kg ip), sacrificed and lungs were excised enbloc. The different lung lobes were cut, blot dried and placed on preweighed glass plates. The wet weight of the tissue was registered immediately. The tray with the tissue was then baked in a hot air oven at 55°C for 72 h to obtain a constant weight. After the dry weight of the tissue was registered, the water content of the tissue was calculated as wet weight minus dry weight and expressed as milligrams of water per milligrams of dry tissue [24].

### 2.6. Transvascular Leakage

The permeability assays were performed by the technique of Heike et al. [25] with minor modifications using sodium fluorescein (Sigma, USA) dye extravasation as an indicator of vascular leakage [26]. After exposure to hypoxia, all the animals received 15 mg/kg bw of sodium fluorescein dye in saline as a bolus through the tail vein and were re-exposed to hypoxia for additional 30 min. The animals were killed and lungs were taken out for the measurement of dye leakage after washing with phosphate-buffered saline to remove the excess dye from the pulmonary vasculature. Lungs were incubated with formamide [27] and the fluorescence was measured after centrifugation at 1500 g for 20 min in the supernatant at an excitation wavelength of 485 nm and emission wavelength of 531 nm using a Varian (USA) spectrofluorimeter. The values are expressed as relative fluorescence units (rfu) per gram dry weight of the tissue.

### 2.7. BALF

Animals were euthanized via anesthetic overdose, a midline thorax to neck incision was made, the ribs removed and a tracheal cannula was placed. The lungs were lavaged in aliquots with sterile phosphate-buffered saline (0.035 mL/g bw). Returned lavage fluid was pooled and centrifuged at 1000 g for 10 min at 4°C for removing cells and debris and the supernatant was aliquoted and frozen at −80°C until assayed.

### 2.8. Total Protein and Albumin Content in the BALF

Total protein in the BALF was measured using commercially available kits obtained from Bangalore Genei, India, whereas albumin levels were estimated using ELISA kit obtained from M/S Shibayage (Japan).

### 2.9. Inflammatory Cytokines in the BALF

Inflammatory cytokines (TNF-*α*, IL-6, IL-10 and MCP-1) in the BALF of different groups of animals were quantified using ELISA kits obtained from M/S BD Biosciences Ltd (USA) as per the procedures provided by the manufacturer and were expressed as pg/ml of BALF.

### 2.10. Oxidative Stress Markers

After exposure to hypoxia the animal's thoracic cavity was opened and the pulmonary vasculature was washed with saline. The lungs were dissected out and immediately stored at −80°C until assayed for oxidative stress parameters.

The lung samples were homogenized with 0.154 M KCl solution in ice cold condition and centrifuged at 3000 g for 15 min at 4°C. The supernatants were analyzed for free radical [28], malonedialdehyde (MDA; [29]), reduced glutathione (GSH; [30]), enzymes like glutathione reductase (GR), glutathione peroxidase (GPx) and superoxide dismutase (SOD) (Randox kits, USA).

### 2.11. Plasma Collection

Following anesthesia the blood samples were collected from the abdominal aorta after opening the abdomen using 20 G heparinized vacutainers (BD). The blood samples were centrifuged immediately at 2500 g for 20 min at 4°C. The plasma samples were then separated and stored at −80°C until assayed.

### 2.12. Plasma Catecholamines

The plasma catecholamines were analyzed in different groups of animals using high performance liquid chromatography (HPLC) system (Waters, USA). The samples for HPLC were prepared by using Chromsystems kit (USA) [31] and the values were expressed in terms of pg/mL of plasma.

### 2.13. BALF and Plasma VEGF

VEGF was quantified in the BALF and plasma by ELISA (R&D Systems, Inc., USA) following instructions of the manufacturer. The concentrations of unknown samples were calculated with reference to the absorbance of VEGF standards and were expressed in terms of pg/mL of BALF.

### 2.14. Immunohistochemical Studies

VEGF expression in lung was also determined by immunohistochemical studies. The lungs were inflated, and the pulmonary artery was perfused with 4% paraformaldehyde. Excised lungs were fixed in 4% paraformaldehyde overnight at 4°C and cryosectioned (Leica, Germany). Frozen sections (60 *μ*m) were used for immunostaining with VEGF-specific antibodies. Sections were treated with 0.3% hydrogen peroxide in methanol for 20 min, preincubated with 5% goat serum and treated with anti-VEGF antibody (1 : 200, Santacruz, USA) for 1 h at 37°C. The sections were then incubated with a biotinylated goat anti-rabbit secondary antibody, treated with the avidin-biotin complex (ABC kit, Santacruz, USA), and stained with diaminobenzidine tetrahydrochloride and hydrogen peroxide.

### 2.15. Vascular Reactivity Studies

The vascular reactivity studies were performed to evaluate vasorelaxant activity of SBT extracts [32]. After 12 h of administration of the SBT extracts, the animals were anaesthetized, thoracic cavity was opened and the lungs enbloc with aorta were removed quickly and placed in cold Kreb's Ringer solution. The main pulmonary artery was dissected free and cleaned of fat and adventitia. The artery was cut into rings of 2-mm long. Each ring was mounted with two L-shaped stainless steel hooks in jacketted tissue chambers containing Kreb's Ringer solution at 37°C and gassed with 95% O_2_ and 5% CO_2_. The upper hook of each aortic ring was attached to a force displacement transducer through a silk suture and changes in isometric force were recorded on a polygraphic system (BIOPAC software, USA). Phenylephrine and acetylcholine were used as standard vasocontractile and vasodilatory agents. Percentage change in the tension during exposure to acetylcholine against the tension of phenylephrine was calculated and compared with various groups for assessing vascular reactivity.

### 2.16. Statistical Analysis

All the values are expressed as mean ± standard deviation (SD). The statistical analysis was done by using one-way ANOVA followed by Tukey test with the help of SPSS software. A value of *P* < .05 was considered as statistically significant.

## 3. Results

### 3.1. Morphological Difference

**Figure 2:**
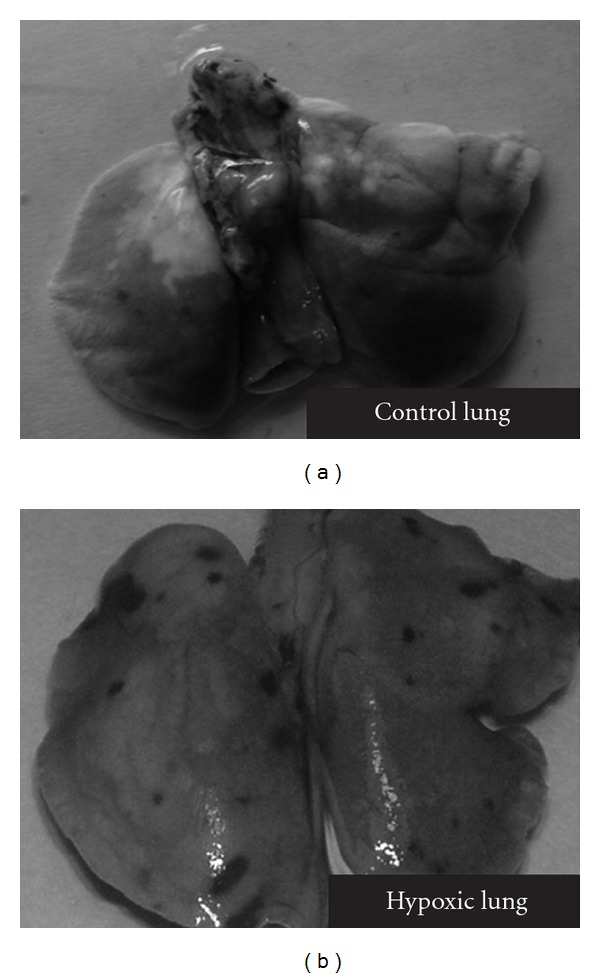
Effect of hypobaric hypoxia on lung morphology. Rats were exposed to stimulated altitude of 9144 m at 24°C for 5 h.

Figure 2 shows the morphological difference between the lungs of normoxic control and hypoxia-exposed animals.

### 3.2. Lung Water Content

**Figure 3:**
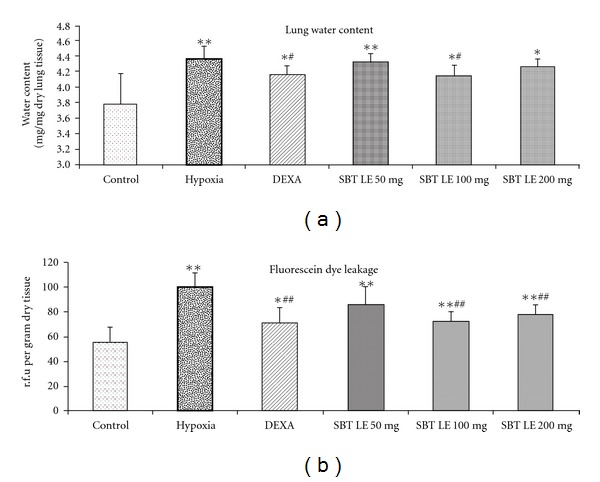
Effect of SBT LE (50, 100 and 200 mg/kg bw) on hypobaric hypoxia-induced vascular permeability. Values are mean ± SD (*n* = 8 per group). Significant test between groups were determined by using one-way ANOVA followed by Tukey test. *P < .05 versus Control; **P < .01 versus Control; ^#^ *P* < .05 versus Hypoxia; ^##^ *P* < .01 versus Hypoxia.

The lung water content in normoxic control animals varied between 3.10 to 4.19 mg/mg dry tissue with a mean of 3.78 ± 0.39 mg/mg dry lung tissue (Figure 3(a)). The mean value of 4.36 ±  0.17 mg/mg dry lung tissue in animals exposed to hypoxia was significantly higher (*P* < .01) than that of normoxic control animals. The lung water content in DEXA and SBT LE (100 mg/Kg bw) treated animals was significantly lower (*P* < .05) than that of hypoxic control animals, but was higher than the normoxic control values.

### 3.3. Transvascular Leakage

The effect of SBT LE on transvascular fluid leakage assessed by quantitation of sodium fluorescein dye leakage is shown in Figure 3(b). In control animals the sodium fluorescein dye leakage varied between 41.46 and 74.55 rfu/g lung tissue with a mean value of 56.81 ± 16.47 rfu/g lung tissue. Following exposure to hypoxia the mean fluorescein dye leakage of 98.97 ± 12.78 rfu/g lung tissue was significantly higher (*P* < .01) as compared with normoxic control values. The animals pretreated with DEXA, which acted as a positive control, showed a significantly lower mean relative fluorescence values (*P* < .01) in lungs (71.39 ± 12.14 rfu/g lung tissue) as compared with hypoxic control animals. Administration of SBT LE (100 and 200 mg/Kg bw) showed a significant decrease (72.81 ± 7.78 and 78.03 ± 7.57 rfu/g lung tissue, respectively) (*P* < .01) in fluorescein dye leakage as compared with hypoxic animals. However, the rfu values in DEXA and SBT LE treated animals were still significantly higher than the normoxic control values. Since 100 mg/Kg bw and 200 mg/Kg bw both provided optimal protection, the lower dose was used for further studies.

### 3.4. Total Protein and Albumin Content in BALF

**Figure 4:**
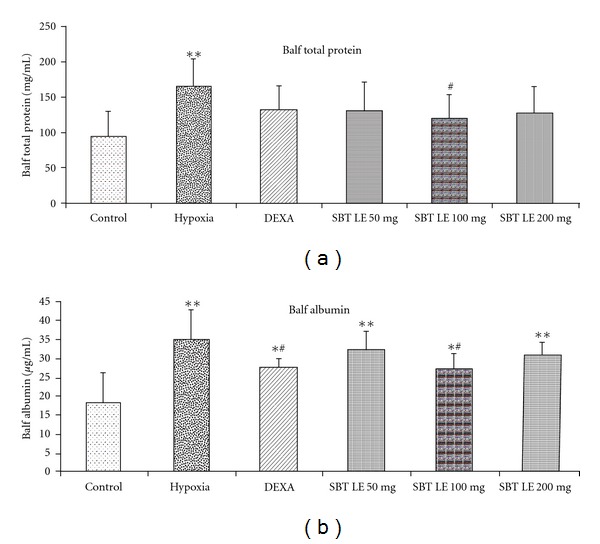
Prevention of extravasation of total protein and albumin by SBT LE (50, 100 and 200 mg/kg bw) in rats exposed to simulated altitude of 9144 m for 5 h. Values are mean ± SD (*n* = 8 per group). Significant test between groups were determined by using one-way ANOVA followed by Tukey test. *P < .05 versus Control; **P < .01 versus Control; ^#^ *P* < .05 versus Hypoxia; ^##^ *P* < .01 versus Hypoxia.

The results of analysis of BALF protein and albumin contents in various groups of animals exposed to hypoxic stress are shown in Figures 4(a) and 4(b). The mean BALF protein content in animals exposed to hypoxia was significantly higher (165.66 ± 43.21 mg/mL) (*P* < .01) as compared with the normoxic control values (93.09 ± 37.95 mg/mL). Pretreatment with DEXA caused a non-significant decline (131.15 ± 34.23 mg/mL) (*P* > .05) in BALF protein contents as compared with the hypoxic animals. In SBT LE treated animals (100 mg/kg b.w.) the BALF protein contents were significantly lower (*P* < .05) (119.11 ± 30.77 mg/mL) as compared with hypoxic animals. In normoxic control animals the albumin contents in BALF varied between 9.25 and 33.17 *μ*g/mL with a mean value of 18.37 ± 8.04 *μ*g/mL. The mean BALF albumin values of 34.67 ± 7.93 *μ*g/mL in animals exposed to hypoxia was significantly higher (*P* < .01) than that of normoxic control values. Administration of SBT LE (100 mg/kg bw) caused a significant decrease (27.35 ± 4.07 *μ*g/mL) (*P* < .05) in BALF albumin contents and the mean value was comparable with DEXA treated animals (27.44 ± 2.54 *μ*g/mL).

### 3.5. Inflammatory Cytokines

**Figure 5:**
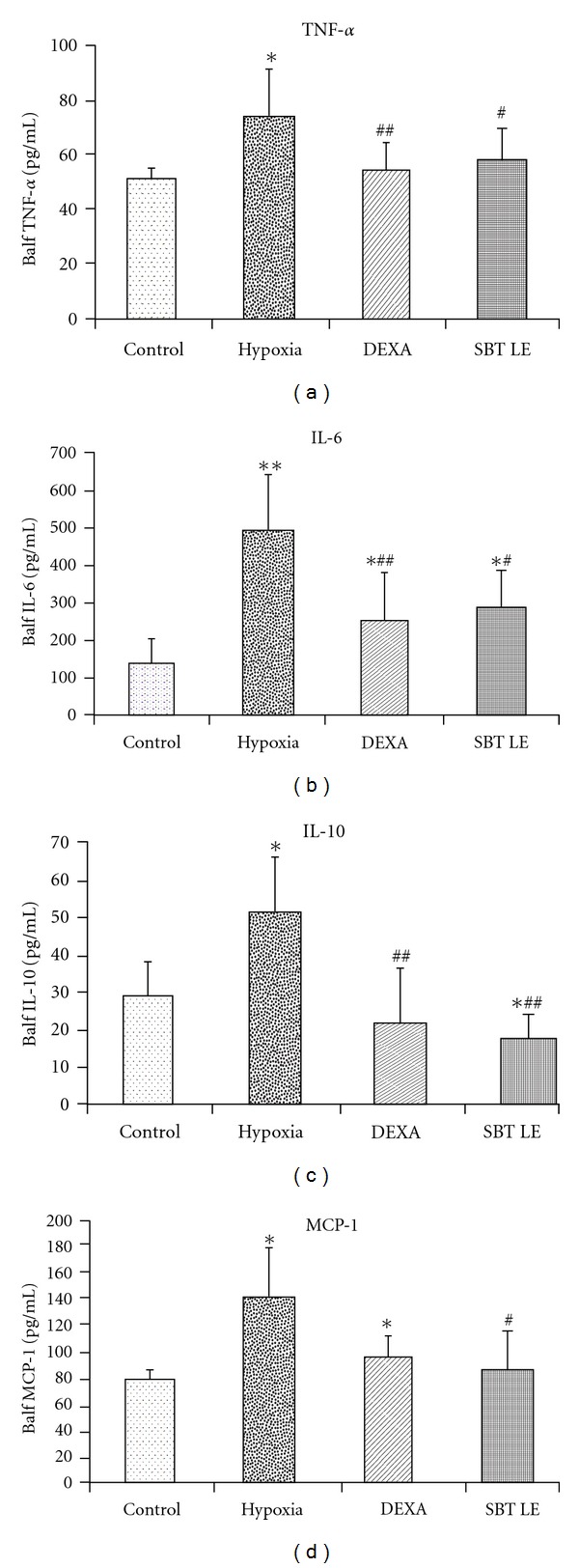
BALF inflammatory cytokines in SBT LE (100 mg/kg bw) treated animals exposed to simulated high altitude of 9144 m for 5 h. Values are mean ± SD (*n* = 8 per group). Significant test between groups were determined by using one-way ANOVA followed by Tukey test. *P < .05 versus Control; **P < .01 versus Control; ^#^ *P* < .05 versus Hypoxia; ^##^ *P* < .01 versus Hypoxia. TNF-*α*, tumor necrosis factor-alpha; IL-6, Interleukin-6; IL-10, Interleukin-10; MCP-1, Macrophage chemoacttractant protein-1.

The cytokines such as TNF-*α*, IL-6, IL-10 and MCP-1 were assayed in the BALF of different groups of animals. Hypoxic exposure caused a significant increase (*P* < .05) in these proinflammatory cytokines—TNF-*α* (44%), IL-6 (260%), IL-10 (76%) and MCP-1 (72%)—in the BALF of animals (Figure 5) than the normoxic controls. However, this hypoxia-induced increase in the proinflammatory cytokines was markedly reduced (*P* < .05) in the animals pretreated with DEXA—TNF-*α* (26%), IL-6 (48%), IL-10 (57%) and MCP-1 (30%)—and SBT LE—TNF-*α* (20%), IL-6 (40%), IL-10 (86%) and MCP-1 (38%).

### 3.6. Oxidative Stress Markers

Hypoxic exposure caused a significant increase (*P* < .05) in the oxidative stress markers like free radicals (87%) and MDA (21%) and marked decrease in GSH (19%) levels in the lung homogenates than the normoxic controls (Table 1, Panel A). The pretreatment of animals with SBT LE significantly attenuated (*P* < .05) the free radical (24%) and MDA (24%) generation and increased the GSH (28%) levels as compared with the hypoxic animals.

Table 1 (Panel B) shows the level of antioxidant enzyme levels such as GR, GPx and SOD analyzed in the lung homogenates of animals of different groups. There was a significant decrease (*P* < .05) in the GR (14%) and GPx (17%) enzyme levels on exposure to hypoxic insult as compared with the normoxic controls. The pretreatment of animals with LE of SBT caused a marked increased in GR (17%) and GPx (16%) levels as compared with the hypoxic animals.

### 3.7. Plasma Catecholamines

Table 2 represents the circulatory levels of catecholamines in different groups of animals. In comparison with the controls, the plasma epinephrine, norepinephrine and dopamine levels were significantly higher when the animals were exposed to hypoxia. The administration of DEXA showed a significant decline (*P* < .05) in the plasma catecholamine levels than the animals exposed to hypoxia. Although, the SBT LE pretreatment showed a marked decline (*P* > .05) in majority of the plasma catecholamine levels than the hypoxic animals, the mean value was not comparable with the DEXA treated animals.

### 3.8. VEGF Level in BALF and Plasma

**Figure 6:**
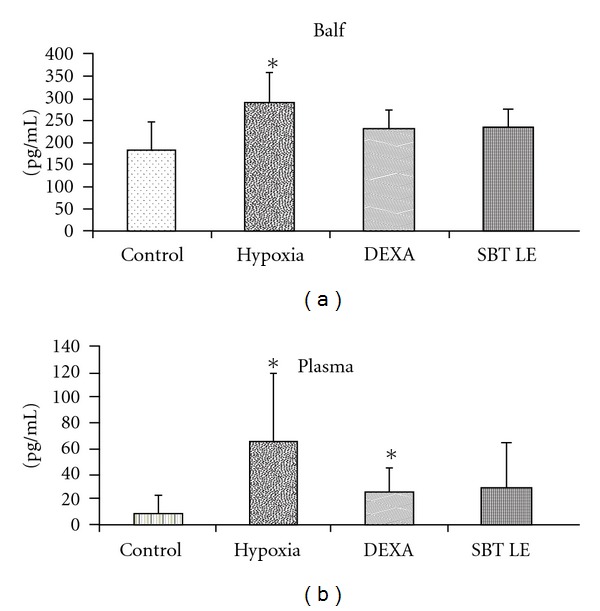
BALF and plasma VEGF levels in control and hypoxic rats pretreated with SBT LE (100 mg/kg bw). Values are mean ± SD (*n* = 8 per group). Significant test between groups were determined by using one-way ANOVA followed by Tukey test. *P < .05 versus Control; **P < .01 versus Control.

In control animals, the BALF VEGF levels varied between 91.14 and 257.22 pg/mL with a mean value of 182.78 ± 62.48 pg/mL (Figure 6(a)). In animals exposed to hypoxia, the mean BALF VEGF value of 289.04 ± 66.38 pg/ml was significantly higher (*P* < 0.05) than the control values. The plasma VEGF levels varied between 0 and 40.90 pg/mL with a mean value of 7.73 ± 13.98 pg/mL. In the animals exposed to hypoxia, the mean value of 64.33 ± 54.89 pg/mL was significantly higher (*P* < .05) than the control values. In SBT LE pretreated animals, although there was a decline in plasma VEGF levels than the animals exposed to hypoxia, the values were not statistically significant (*P* > .05) (Figure 6(b)).

### 3.9. Lung VEGF Immunohistochemistry

**Figure 7:**
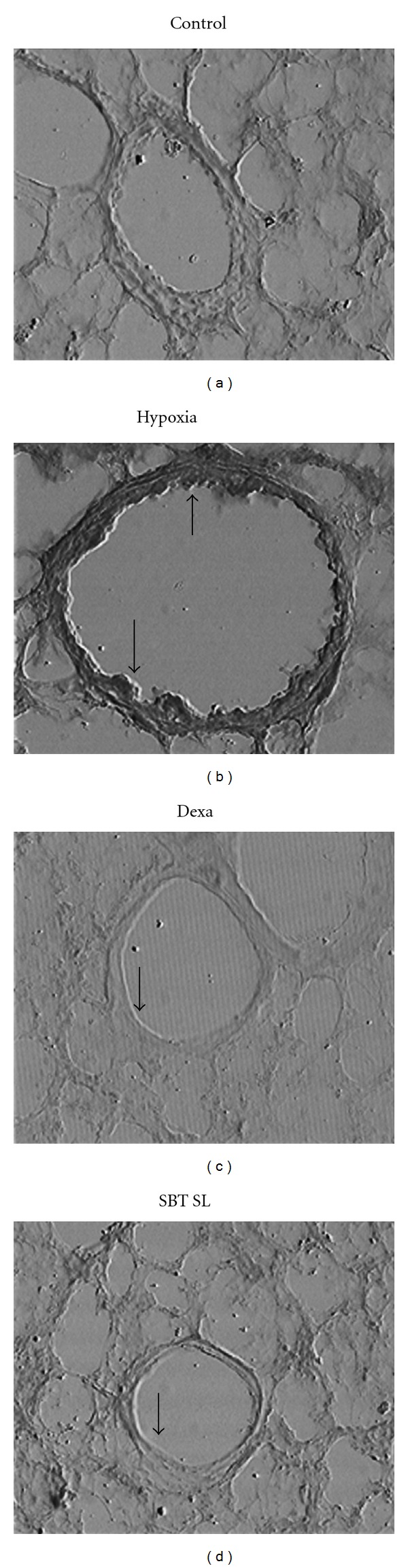
Reduction of VEGF immunohistochemical staining after pretreatment with DEXA and SBT LE (100 mg/kg bw) in rats exposed to simulated high altitude of 9144 m for 5 h. Minimal VEGF immunostaining was evident in lung parenchyma in control animals in contrast to abundant VEGF immunostaning in the hypoxic group. Pretreatment with DEXA, SBT LE reduced VEGF staining and maintained the capillary integrity. The photographs are representative of three animals examined under each condition. Original magnification: ×200.

VEGF immunoreactivity was detected throughout the lung parenchyma after 5 h of hypoxic exposure (Figure 7). As evident from the Figure 7 low degree of staining was observed in the lungs of control animals. In contrast, higher VEGF immunoreactivity was present in alveolar epithelial cells, vascular structures, as well as in large airways of hypoxic animals. The VEGF immunostaining in lung parenchyma of the animals pretreated with DEXA, SBT LE was markedly decreased as compared with the group exposed to hypoxia only.

### 3.10. Vascular Reactivity Studies

**Figure 8:**
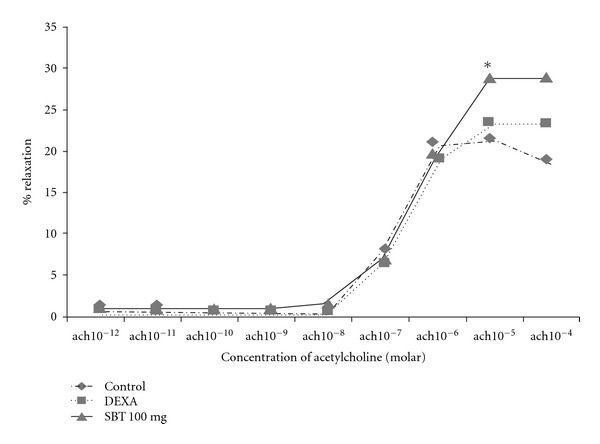
Effect of SBT LE on vascular reactivity. The animals were pretreated with DEXA and SBT LE (100 mg/kg bw). Vascular reactivity response in control and treated groups. Significant test between groups were determined by using one-way ANOVA followed by Tukey test. *P < .05 versus control. Ach, acetylcholine.

Figure 8 shows the vascular reactivity of pulmonary arterial segments of animals in different groups. Addition of phenylephrine 10^*‒*6^ M caused a significant vasoconstriction that could be relieved by addition of acetylcholine in a dose-dependent manner. In the control animals, the optimum acetylcholine concentration dependent relaxation was observed at 10^*‒*5^ M (21.30 ± 9.41%). On pretreatment of animals with SBT LE, a significant increase (*P* < .05) in acetylcholine concentration dependent relaxation (28.48 ± 5.86%) was observed in comparison with the control animals which was 25% higher than the control values.

## 4. Discussion

The results from the present study suggest that SBT LE is able to enhance hypoxic tolerance in experimental animals subjected to simulated hypobaric hypoxic stress. Administration of SBT LE caused a marked decline in transvascular fluid leakage into the lungs besides curtailing leakage of proteins and albumin into the alveoli and decreasing the proinflammatory markers in the lung.

HAPE has been demonstrated to occur due to increased leakage of vascular components into the lungs as a result of hypoxic insult. Many previous studies have shown that HAPE is associated with increase in leakage of fluids, proteins like albumin and other vascular components [2, 33–35] from the capillaries into the alveolar space.

Our observations on increase in lung water content following exposure to hypoxia are in agreement with the observations of earlier investigators [34, 36]. The increase in lung water content appears to be due to leakage of fluids from intravascular to extravascular fluid compartment [37, 38]. However, when SBT pretreated animals were exposed to hypoxia, a significant decline in lung water content was observed as compared with hypoxic controls, which was at par with DEXA treated animals. Besides measuring the lung water content, the transvascular fluid leakage was also monitored using sodium fluorescein dye as an indicator. Animals exposed to hypoxia showed a significant increase in fluorescein dye leakage as compared with normoxic controls. This observation coincides with the findings of Heike et al. [25] who have demonstrated an increase in fluorescein dye leakage in the brain blood vessels, as an indicator of enhanced vascular permeability in mice exposed to hypoxic stress. The observation that SBT LE was able to curtail the dye leakage following exposure to hypoxia suggests that the extract is able to maintain alveolar arterial capillary membrane integrity.

Many earlier studies have reported that HAPE is associated with the leakage of highly concentrated proteinaeous fluid [36, 38, 39] specifically albumin [40–43] into the lung from the pulmonary vasculature. The results of our study also demonstrated a marked increase in total protein and albumin in the BALF of animals exposed to hypoxic stress. However, administration of SBT LE significantly curtailed hypoxia-induced increase in total protein and albumin leakage suggesting that the LE besides decreasing fluid leakage is also able to inhibit leakage of proteins into the alveoli.

To evaluate the role of lung inflammation in setting up the pathogenesis of HAPE under decreased alveolar oxygen levels, proinflammatory cytokines such as TNF-*α*, IL-6, IL-10 and MCP-1 were also measured in the BALF of animals subjected to hypoxic stress since all these cytokines have been suggested to be associated with airway inflammation in HAPE patients [40]. Hypoxic exposure caused a significant increase in the inflammatory interleukins (TNF-*α*, IL-6, IL-10 and MCP-1) as compared with the normoxic controls. However, the animals pretreated with SBT extract showed a marked decrease in these interleukins as compared with hypoxic controls suggesting that hypoxia-induced alveolar inflammation can be prevented significantly by SBT LE.

Besides curtailing hypoxia-induced alveolar inflammation SBT LE also caused a marked reduction in alveolar oxidative stress. The reactive oxygen and nitrogen species have been implicated in the causation of AMS, HAPE and HACE [5, 44–46]. Free radicals are implicated to damage biomembranes, thereby compromising cell integrity and function [47]. Besides increasing pulmonary arterial pressure [4], the free radical production under hypoxic environment may cause oxidative injury of the endothelium [48], resulting in increased pulmonary capillary permeability. Hence, the oxidative stress markers (free radical, GSH, MDA, SOD, GPx, GR) were measured in the lung homogenates of different groups of animals. Hypoxic exposure caused a significant increase in the levels of free radical and MDA and decreased the antioxidant enzyme system. Pretreatment of animals with SBT extracts about 12 h prior to hypoxic exposure caused a marked reduction in free radical and MDA levels and increased GSH, SOD, GPx and GR, thereby providing a significant protection against hypoxia-induced oxidative damage.

Acute hypoxemia has been shown to enhance sympathetic nerve activity, release of catecholamines, regional vasoconstriction and heart rate in spontaneously breathing and anaesthetized mammals [49, 50]. The catecholamines have been also demonstrated to augment the production of free radicals by auto-oxidation of monoamines to produce superoxide and hydrogen peroxide radicals under hypoxic stress [50, 51]. Therefore, the circulatory levels of catecholamines were estimated in animals exposed to hypoxic stress. The epinephrine, norepinephrine and dopamine levels were markedly increased in animals following exposure to hypoxia. However, this rise in plasma epinephrine, norepinephrine and dopamine in animals treated with SBT LE was significantly lower than in the untreated animals.

VEGF is a potent mediator of capillary leak if it gains access to its receptors on the capillary endothelium and may be influenced by hypoxic stress. Over expression of VEGF has been demonstrated to induce formation of fenestrations (small pores) and the thin endothelial cell cytoplasm may allow leak of solutes [53]. Therefore, VEGF estimation was carried out in plasma as well as in BALF of animals after exposure to hypoxic stress. In the BALF of the hypoxic animals, the VEGF levels were found to be 58% higher than the controls. However, pretreatment with SBT LE inhibited hypoxia-induced VEGF increase in the BALF by 18% as compared with the hypoxic group. In plasma, the VEGF levels of control animals were almost undetectable. There was a marked increase in circulatory levels of VEGF following exposure to hypoxia. However, this increase in plasma VEGF was attenuated when the animals were pretreated with SBT LE (57%). Although blood levels do not generally reflect the pulmonary concentrations, our immunohistochemistry results confirmed the abundant presence of VEGF peptide in the lung parenchyma in the setting of hypoxia. The staining of VEGF was very intense in the alveolar parenchyma in the animals exposed to hypoxic stress than the controls. However, a marked reduction in the immunohistochemical staining was observed when the animals were pretreated with SBT LE as compared with hypoxic controls.

The vascular reactivity studies were carried out in the aortic rings of different groups of animals to evaluate the vasorelaxant activity of SBT LE. As compared with controls, the SBT LE pretreated group showed a significant vasorelaxation response. The vasorelaxant activity of the SBT leaf may be due to the presence of ß-Amyrinoleylalcohol acid, which has been reported to dilate the cardiac and cerebral vessels, thereby facilitating blood circulation and lowering of the blood pressure [54]. Since hypoxia-induced alveolar vasoconstriction has been suggested as an important contributory factor in the pathogenesis of HAPE, the LE may counteract vasocontractile effect of the hypoxic stress.

**Figure 9:**
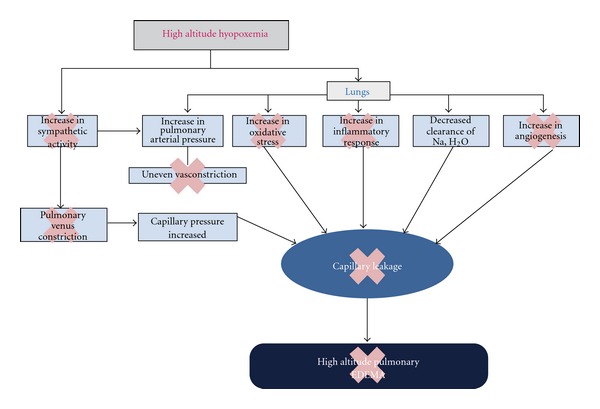
Mechanism of prevention of SBT LE against hypoxia-indused vascular leakage. The cross indicates that SBT LE is able to inhibit the hypoxia-induced pathophysiological process, thereby inhibiting the capillary leakage.

Hence from the above observations, the SBT LE provided significant protection against hypoxia-induced transvascular permeability by reducing alveolar oxidative stress and inflammation. The extract also attenuated the hypoxia-induced increase in VEGF and catecholamine levels, which in turn was responsible for a decrease in transvascular fluid leakage in the lungs (Figure 9). The main antioxidant components of leaves are flavonoids, which comprises of leucoanthocyanidins, quercetin, isorhamnetin, epicatechin and flavonols [55]. Therefore, it is possible that SBT LE by enhancing the endogenous antioxidant defense system and by curtailing the oxidative stress and inflammatory response, may maintain the alveolar membrane integrity and thus prevent hypoxia-induced increase in alveolar vascular permeability.

In conclusion, these results suggest that HAPE whose pathogenesis is a multifactorial phenomenon, can be prevented significantly by the diverse activities of bioactive molecules present in SBT LE, which can be developed as food supplement and/or nutrceutical to enhance tolerance to the hypoxic environment.

## Figures and Tables

**Table 1:** Effect of SBT LE (100 mg/kg bw) on changes in hypobaric hypoxia-induced oxidative stress markers and antioxidant enzymes in lungs of different groups of animals.

	Control	Hypoxia	DEXA 5 mg/kg bw	SBT LE 100 mg/kg bw
Panel A				
Rfu	4.03 ± 0.99	7.57 ± 1.79**	6.63 ± 1.52**	5.76 ± 0.64^∗#^
MDA (n mol/g)	170.6 ± 19.8	211.8 ± 13.0**	174.1 ± 25.5^#^	159.4 ± 19.5^##^
Panel B				
GSH (ng/g)	19.2 ± 1.6	15.4 ± 3.3*	17.5 ± 4.9	19.7 ± 4.1^#^
GR (U/L)	190.59 ± 18.12	162.57 ± 10.02**	185.75 ± 25.05	190.60 ± 5.01^##^
GPx (U/L)	781.38 ± 69.61	644.55 ± 53.50*	663.14 ± 32.41*	744.92 ± 38.17^#^
SOD (U/mL)	4.16 ± 0.30	3.96 ± 0.31	3.97 ± 0.35	4.07 ± 0.17

Values are expressed as mean ± SD (*n* = 8 per group). Significant test between groups were determined by using one-way ANOVA followed by Tukey test.

*P < .05 versus Control; **P < .01 versus Control; ^#^ *P* < .05 versus hypoxia; ^##^ *P* < .01 versus hypoxia.

**Table 2:** Circulating levels of epinephrine, norepinephrine and dopamine in animals pretreated with DEXA, SBT LE (100 mg/kg bw) following exposure to hypoxia of 9144 m for 5 h.

	Control	Hypoxia	DEXA 5 mg/kg bw	SBT SL 100 mg/kg bw
Epinephrine (pg/mL)	122.07 ± 90.31	956.92 ± 884.12**	406.96 ± 361.17^∗#^	739.46 ± 490.22**
Norepinephrine (pg/mL)	103.23 ± 67.74	1201 ± 880.14**	296.30 ± 168.71^∗∗##^	781.85 ± 528.38^∗∗#^
Dopamine (pg/mL)	34.46 ± 28.45	1508.44 ± 716.25*	138.84 ± 406.46^##^	460.15 ± 348.58^∗#^

Values are expressed as mean ± SD (*n* = 16 per group). Significant test between groups were determined by using one-way ANOVA followed by Tukey test.

*P < .05 versus Control; **P < .01 versus Control; ^#^ *P* < .05 versus hypoxia; ^##^ *P* < .01 versus hypoxia.

## References

[1] Hackett PH, Roach RC, Auerbach PS (2001). High-altitude medicine. *Wilderness Medicine*.

[2] Hultgren HN (1996). High-altitude pulmonary edema: current concepts. *Annual Review of Medicine*.

[3] Bärtsch P, Mairbäurl H, Swenson ER, Maggiorini M (2003). High altitude pulmonary oedema. *Swiss Medical Weekly*.

[4] Hoshikawa Y, Ono S, Suzuki S (2001). Generation of oxidative stress contributes to the development of pulmonary hypertension induced by hypoxia. *Journal of Applied Physiology*.

[5] Bakonyi T, Radak Z (2004). High altitude and free radicals. *Journal of Sports Science and Medicine*.

[6] Madjdpour C, Jewell UR, Kneller S (2003). Decreased alveolar oxygen induces lung inflammation. *American Journal of Physiology—Lung Cellular and Molecular Physiology*.

[7] Leung DW, Cachianes G, Kuang W-J, Goeddel DV, Ferrara N (1989). Vascular endothelial growth factor is a secreted angiogenic mitogen. *Science*.

[8] Ferrara N, Houck K, Jakeman L, Leung DW (1992). Molecular and biological properties of the vascular endothelial growth factor family of proteins. *Endocrine Reviews*.

[9] Collins PD, Connolly DT, Williams TJ (1993). Characterization of the increase in vascular permeability induced by vascular permeability factor *in vivo*. *British Journal of Pharmacology*.

[10] Minchenko A, Bauer T, Salceda S, Caro J (1994). Hypoxic stimulation of vascular endothelial growth factor expression *in vitro* and *in vivo*. *Laboratory Investigation*.

[11] Helen C, Atsushi Y, Victoria A, Toshisuke M, Stella K (1998). Increased vascular endotheialial growth factor production in the lungs of rats with hypoxia-induced pulmonary hypertension. *American Journal of Respiratory Cell and Molecular Biology*.

[12] Kaner RJ, Ladetto JV, Singh R, Fukuda N, Matthay MA, Crystal RG (2000). Lung overexpression of the vascular endothelial growth factor gene induces pulmonary edema. *American Journal of Respiratory Cell and Molecular Biology*.

[13] Masatoshi K, Emile EV, Shiro A (1996). Reactive oxygen intermediates increase vascular endothelial growth factor expression *in vitro* and *in vivo*. *Journal of Clinical Investigation*.

[14] Lelkes PI, Hahn KL, Sukovich DA, Karmiol S, Schmidt DH (1998). On the possible role of reactive oxygen species in angiogenesis. *Advances in Experimental Medicine and Biology*.

[15] Xu MY, Sun XX, Tong WX (1994). Medical research and development of Seabuckthorn. *Hippophae*.

[16] Beveridge T, Li TSC, Oomah BD, Smith A (1999). Sea buckthorn products: manufacture and composition. *Journal of Agricultural and Food Chemistry*.

[17] Alam Z (2004). Important therapeutic uses of seabuckthorn (*Hippophae*): a review. *Journal of Biological Sciences*.

[18] Geetha S, Sairam M, Singh V, Ilavazhagan G, Sawhney RC (2002). Anti-oxidant and immunomodulatory properties of seabuckthorn (*Hippophae rhamnoides*)—an *in vitro* study. *Journal of Ethnopharmacology*.

[19] Narayanan S, Ruma D, Gitika B (2005). Antioxidant activities of seabuckthorn (*Hippophae rhamnoides*) during hypoxia induced oxidative stress in glial cells. *Molecular and Cellular Biochemistry*.

[20] Gupta A, Kumar R, Pal K, Banerjee PK, Sawhney RC (2005). A preclinical study of the effects of seabuckthorn (*Hippophae rhamnoides* L.) leaf extract on cutaneous wound healing in albino rats. *International Journal of Lower Extremity Wounds*.

[21] Geetha S, Sairam M, Mongia SS, Virendra S, Ilavazhagan G, Sawhney RC (2003). Evaluation of antioxidant activity of leaf extract of seabuckthorn (*Hippophae rhamnoides* L.) on chromium (VI) induced oxidative stress in albino rats. *Journal of Ethnopharmacology*.

[22] Lilly G, Yogendra P, Richa S (2005). Anti-inflammatory activity of Seabuckthorn (*Hippophae rhamnoides* L.) leaves. *International Immunopharmacology*.

[23] Saggu S, Divekar HM, Gupta V, Sawhney RC, Banerjee PK, Kumar R (2007). Adaptogenic and safety evaluation of seabuckthorn (*Hippophae rhamnoides*) leaf extract: a dose dependent study. *Food and Chemical Toxicology*.

[24] Shelley XLZ, James JM, David G, Yang W (2004). Whole-body hypoxic preconditioning protects mice against acute hypoxia by improving lung function. *Journal of Applied Physiology*.

[25] Schoch HJ, Fischer S, Marti HH (2002). Hypoxia-induced vascular endothelial growth factor expression causes vascular leakage in the brain. *Brain*.

[26] Jayamurthy P, Geetha S, Dhananjay S (2008). Modulatory effects of seabuckthorn (*Hippophae rhamnoides* L.) in hypobaric hypoxia induced cerebral vascular injury. *Brain Research Bulletin*.

[27] Jacob JSH, Rosen ES, Young E (1982). Report on the presence of a toxic substance, dimethyl formamide, in sodium fluorescein used for fluorescein angiography. *British Journal of Ophthalmology*.

[28] Cathcart R, Schwiers E, Ames BN (1983). Detection of picomole levels of hydroperoxides using a fluorescent dichlorofluorescein assay. *Analytical Biochemistry*.

[29] Dousset JC, Trouillh M, Fogliettis MJ (1983). Plasma malondialdehyde levels during myocardial infarction. *Clinica Chimica Acta*.

[30] Kum-Tatt L, Tan IK (1974). A new colorimetric method for the determination of glutathione in erythrocytes. *Clinica Chimica Acta*.

[31] Kienbaum P, Heuter T, Michel MC, Scherbaum N, Gastpar M, Peters J (2001). Chronic *μ*-opioid receptor stimulation in humans decreases muscle sympathetic nerve activity. *Circulation*.

[32] Basu M, Prasad R, Jayamurthy P, Pal K, Arumughan C, Sawhney RC (2007). Anti-atherogenic effects of seabuckthorn (*Hippophaea rhamnoides*) seed oil. *Phytomedicine*.

[33] Peacock AJ (1995). High altitude pulmonary oedema: who gets it and why?. *European Respiratory Journal*.

[34] Carpenter TC, Stenmark KR (2001). Hypoxia decreases lung neprilysin expression and increases pulmonary vascular leak. *American Journal of Physiology—Lung Cellular and Molecular Physiology*.

[35] Carpenter TC, Schomberg S, Stenmark KR (2005). Endothelin-mediated increases in lung VEGF content promote vascular leak in young rats exposed to viral infection and hypoxia. *American Journal of Physiology—Lung Cellular and Molecular Physiology*.

[36] Berg JT (2004). Ginkgo biloba extract prevents high altitude pulmonary edema in rats. *High Altitude Medicine and Biology*.

[37] Schoene RB, Swenson ER, Hultgren HN, Thomas FH, Robert BS (2001). High-altitude pulmonary edema. *High Altitude; An Exploration of Human Adaptation*.

[38] Lorraine BW, Michael AM (2005). Acute Pulmonary Edema. *New England Journal of Medicine*.

[39] Bartsch P (1997). High altitude pulmonary edema. *Respiration*.

[40] Keshi K, Masayuki H, Toshihide H, Takashige M, Tsutomu H, Muneharu H (1998). Inflammatory cytokines in BAL fluid and pulmonary hemodynamics in high-altitude pulmonary edema. *Respiratory Physiology*.

[41] Swenson ER, Maggiorini M, Mongovin S (2002). Pathogenesis of high-altitude pulmonary edema: inflammation is not an etiologic factor. *Journal of the American Medical Association*.

[42] Urano T, Kuwahira I, Iwamoto T (2005). Exposure to hypoxia results in uneven pulmonary blood flow distribution prior to pulmonary edema. *Tokai Journal of Experimental and Clinical Medicine*.

[43] Dehler M, Zessin E, Bärtsch P, Mairbäurl H (2006). Hypoxia causes permeability oedema in the constant-pressure perfused rat lung. *European Respiratory Journal*.

[44] Bailey DM, Davies B (2001). Acute mountain sickness; prophylactic benefits of antioxidant vitamin supplementation at high altitude. *High Altitude Medicine and Biology*.

[45] Baumgartner RW, Bärtsch P (2002). Ataxia in acute mountain sickness does not improve with short-term oxygen inhalation. *High Altitude Medicine and Biology*.

[46] Chao W-H, Askew EW, Roberts DE, Wood SM, Perkins JB (1999). Oxidative stress in humans during work at moderate altitude. *Journal of Nutrition*.

[47] Vanita G, Asheesh G, Shalini S, Harish MD, Grover SK, Ratan K (2005). Anti-stress and adaptogenic activity of L-arginine supplementation. *Evidence-based Complementary and Alternative Medicine*.

[48] Herget J, Wilhelm J, Novotná J (2000). A possible role of the oxidant tissue injury in the development of hypoxic pulmonary hypertension. *Physiological Research*.

[49] Heistad DD, Abboud FM (1980). Circulatory adjustments to hypoxia. *Circulation*.

[50] Rowell LB, Blackmon JR (1986). Lack of sympathetic vasoconstriction in hypoxemic humans at rest. *American Journal of Physiology—Heart and Circulatory Physiology*.

[51] Rathore N, John S, Kale M, Bhatnagar D (1998). Lipid peroxidation and antioxidant enzymes in isoproterenol induced oxidative stress in rat tissues. *Pharmacological Research*.

[52] Jefferson JA, Simoni J, Escudero E (2004). Increased oxidative stress following acute and chronic high altitude exposure. *High Altitude Medicine and Biology*.

[53] Roberts WG, Palade GE (1995). Increased microvascular permeability and endothelial fenestration induced by vascular endothelial growth factor. *Journal of Cell Science*.

[54] Xiaoyan G (1986). Preliminary research on the chemical composition of seabuckthorn. *Journal of Chinese Herbs*.

[55] Tolkachev ON, Sheichenko OP, Singh V (2006). Flavonoids of seabuckthorn (*Hippophae rhamnoides* L.): chemistry and pharmacology. *Seabuckthorn (*Hippophae* L.) A multipurpose Wonder Plant*.

